# A new species of *Habralictus* Moure from Dominica, Lesser Antilles (Hymenoptera, Halictidae)

**DOI:** 10.3897/zookeys.168.2524

**Published:** 2012-01-31

**Authors:** Jason Gibbs

**Affiliations:** 1 Cornell University, Entomology Department, 3119 Comstock Hall, Ithaca, New York, USA

**Keywords:** *Habralictus*, Caenohalictini, Halictinae, Halictidae, Apoidea, taxonomy, Dominica, Lesser Antilles

## Abstract

A new species of *Habralictus* Moure (Apoidea, Halictidae) is described from the island of Dominica, *Habralictus gonzalezi* **sp. n.** The species is distinguished from other West Indian *Habralictus* and a key is provided to the West Indian *Habralictus*. Brief comments on the genus *Habralictus* and bee species of Dominica are provided.

## Introduction

*Habralictus* Moure is a genus of small Neotropical bees known from southern Brazil north to the Jalisco province of Mexico (Michener 2007). *Habralictus* is known to form solitary and communal underground nests (Michener and Lange 1958; Michener et al. 1979). Twenty-four species have been described (Moure 2007; Smith-Pardo 2009) but more species undoubtedly occur on the mainland (Smith-Pardo 2009). Only two species have been reported from the Lesser Antilles, *Habralictus claviventris* (Ashmead, 1900) from St. Vincent and the Grenadines and *Habralictus insularis* Smith-Pardo (2009) from Grenada.

*Habralictus* belongs to the halictid tribe Caenohalictini (or Caenohalictinaof Halictini *sensu lato*), which is composed mostly of large-bodied Neotropical genera (Roberts and Brooks 1987; Michener 2007). *Habralictus* can be distinguished from other halictines by the following combination of characters: size small (4.0–6.5 mm), head and mesosoma bright iridescent green to blackish (usually with metallic tints), fore wing with strong distal veins (i.e. 1rs-m, 2r-sm), female metafemur with scopa, metasoma terga without apical or basal hair bands (females often with yellow maculations), posterior surface of propodeum not enclosed by carinae, inner metatibial spur pectinate (usually ciliate in males), dorsal surface of propodeum (including metapostnotum) with long horizontal portion, eyes bare or with short setae. Male *Habralictus* have metasoma petiolate.

Two subgenera of *Habralictus* have been recognised in the past, *Habralictus sensu stricto* and *Zikaniella* Moure(see Michener 2007). The latter seems to render the former paraphyletic (Gonçalves and Melo 2010) so *Zikaniella* should be considered a junior synonym (*Habralictus* has precedence; Michener 2007). *Habralictus insularis* was reported to have characteristics of both nominal subgenera (Smith-Pardo 2009), but this was apparently an error due to the application of female characters of *Habralictus insularis* (i.e. the pectinate inner metatibial spur) to the subgeneric key, which is based solely on males (Michener 2007).

A new species of *Habralictus* is described from material collected in the Commonwealth of Dominica. This species is the most northern representative of *Habralictus* in the Lesser Antilles and only the fourth halictid bee known from Dominica (Crawford 1914; Eickwort and Stage 1972).

## Methods

Descriptions are modified from a format used for other halictid bees (e.g.Gibbs 2010, 2011). Terminology for structures follows that of Engel (2001, 2009), and Michener (2007) and for sculpturing that of Harris (1979). The following abbreviations are used in the descriptions: upper ocular distance (UOD), lower ocular distance (LOD), and lateral ocellar diameter (OD; used as a relative measure for hair length). Flagellomeres and metasomal terga and sterna are referred to by “F”, “T”, and “S” followed by the appropriate number. Specimens examined were deposited at BBSL, Bee Biology and Systematics Laboratory, USDA-ARS, Utah State University, Logan, Utah, USA. Individual paratypes will be deposited at other major bee collections including the AMNH, American Museum of Natural History, New York, New York, USA; CUIC, Cornell University Insect Collection, Ithaca, New York, USA; National Museum of Natural History, Washington D.C., USA; PCYU, Packer Collection York University, Toronto, Ontario, Canada; and SEMC, Snow Entomological Museum, Lawrence, Kansas, USA.

Measurements were taken using an ocular micrometer in a Zeiss Stemi SV 6 microscope (Oberkochen, Germany) and by examining Figs 2, 3, 8 and 9 using Adobe Photoshop CS5 (Adobe Systems Inc.). Measurements of the head were taken in frontal view (as in Figs 2, 8). Head length was measured medially from the vertex to the distal margin of the clypeus. Head width was measured from the outer margins of the compound eyes. UOD and LOD were taken to be the minimum distance between inner margins of the compound eyes, measured from above and below the eye emargination, respectively. The supraclypeal area was measured from the lower margin of the antennal sockets to the upper margin of the clypeus. The width of the mesosoma was measured between the outer margins of the pronotal lobes in dorsal view. The lengths of the mesoscutellum and dorsal propodeal surface were measured medially in dorsal view.

## Systematics

### Genus HabralictusMoure

*Habralictus* Moure 1941: 59. Type species: *Habralictus flavopictus* Moure 1941, by original designation.

*Zikaniella* Moure 1941: 57. Type species: *Zikaniella crassiceps* Moure 1941, by original designation and monotypy.

#### Habralictus gonzalezi sp. n.

urn:lsid:zoobank.org:act:543C0A86-FA4A-434F-B873-1EBBB1BF0395

http://species-id.net/wiki/Habralictus_gonzalezi

##### Type material.

*Holotype* ♀: DOMINICA: Parish of St. Joseph, Springfield Estate, 13.34667°N, 61.3683°W, 430 m, 15–20 Mar. 2003, (M. E. Irwin, M. Shepard), Malaise trap [BBSL]. *Allotype* ♂: topotypical [BBSL]. 10 *paratype* ♀♀: topotypical [AMNH, BBSL (5), CUIC, NMNH, PCYU, SEMC].

##### Diagnosis.

Females of *Habralictus gonzalezi* can be distinguished from *Habralictus insularis* by the following: face mostly green (mostly copper in *Habralictus insularis*), clypeus with few punctures limited to medial area (numerous punctures throughout in *Habralictus insularis*), supraclypeal area virtually impunctate (distinctly punctate in *Habralictus insularis*), pronotal lobe dark brown (yellow-orange in *Habralictus insularis*), and metatibial anterobasal hairs brown (off-white in *Habralictus insularis*).

Males of *Habralictus gonzalezi* can be distinguished from *Habralictus insularis* and *Habralictus claviventris* by the following: supraclypeal area and lower paraocular area imbricate, punctures obscure (smooth, distinctly punctate in *Habralictus claviventris* and *Habralictus insularis*);mesoscutum and mesoscutellum without evident punctation (punctures present albeit fine in *Habralictus claviventris* and *Habralictus insularis*); head and mesosoma bluish-green (bright green in *Habralictus claviventris* and *Habralictus insularis*).

##### Description.

**Figure 1.**
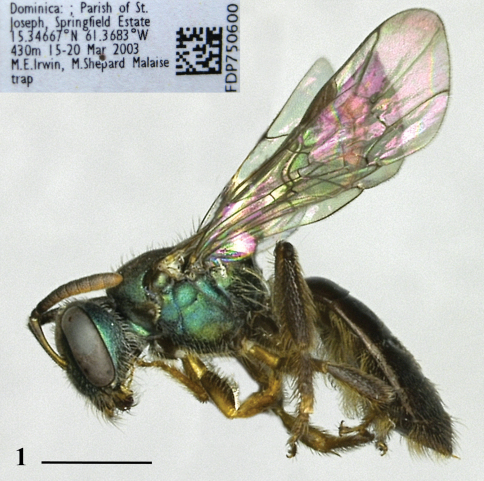
Holotype female of *Habralictus gonzalezi* sp. n. in lateral view with locality label (inset). Scale bar = 1 mm.

**Figures 2–7.**
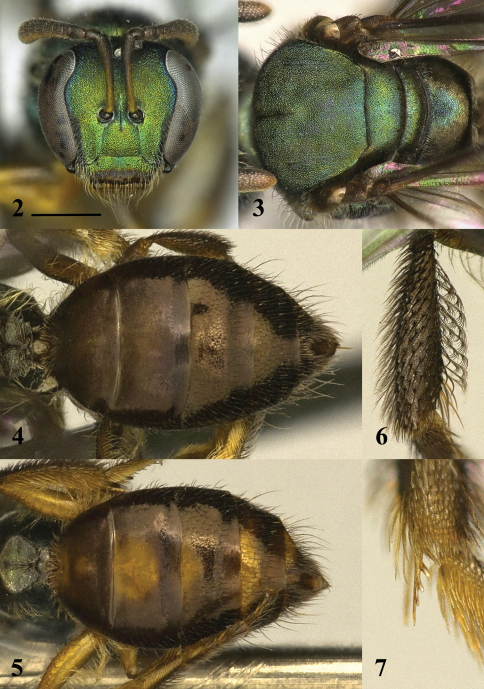
Female of *Habralictus gonzalezi* sp. n. **2** Face (holotype), scale bar = 0.5 mm **3** Dorsal view of mesosoma (holotype) **4** dorsal view of metasoma (holotype) **5** dorsal view of metasoma (paratype) **6** anterior view of metatibia (paratype) **7** inner metatibial spur (paratype).

*Female.* (Figs 1–7). Body length: 3.5–4. 3 mm. Head length: 1.04–1.14 mm. Head width: 1.10–1.24 mm. Forewing length: 3.0–3.1 mm.

***Structure.*** **Head:** Face wider than long (length/width ratio = 0.92–0.94). Eyes weakly convergent below (UOD:LOD = 1.03–1.07). Clypeus shorter than length of supraclypeal area (ratio = 1.06–1.13). Mandible with preapical tooth. Labrum without distinct basal elevation; apical process with dorsoapical keel. Scape slender, weakly clavate; extending above slightly above lateral ocelli. Pedicel subequal to F1 and F2 combined. F1 and F3 both shorter than F2. Flagellum clavate. Preoccipital area rounded. Gena narrower than eye. **Mesosoma:** Pronotum with dorsolateral ridge broadly rounded, interrupted by transverse sulcus; dorsolateral angle low, obtuse, indistinct. Mesoscutum subequal in width to head; anterior margin raised steeply above pronotum; parapsidal line fine, somewhat obscure. Mesoscutellum flat, without medial depression. Episternal groove below scrobe curving sharply and widening towards anterior. Lateral surface of procoxa concave. Inner metatibial spur pectinate with four branches (not including apex of rachis). Tegula ovoid, slightly narrowed anteriorly. Marginal cell narrow towards apex; free portion 3× length of portion subtended by submarginal cells. Distal hamuli arranged 2-1-2 (approaching 2–3). Dorsal surface of propodeum (including metapostnotum) longer than mesoscutellum (ratio = 1.07–1.09); posterior margin of dorsal surface rounded. Posterior surface of propodeum slightly concave; lateral carina fine, reaching two thirds distance to dorsal surface. **Metasoma:** Ovoid, flat; terga, especially T1–T3 with lateral portions sharply reflexed ventrally. Area beyond premarginal line weakly impressed.

***Colour.*Head:** Mostly green; face with golden and coppery reflections. Labrum and lower half of clypeus dark brown. Mandible brownish yellow except apex red. Antenna dark brown, except lateral surface of scape dull yellow and ventral surface of flagellum orange-yellow. Vertex dark green-blue. **Mesosoma:** Dark green dorsally, lighter ventrally. Pronotal lobe dark brown. Ventral half of mesepisternum golden or brassy. Fore leg yellow, except profemur on dorsal half (sometimes) and ventrally. Mid leg dark brown, except protrochanter and posterior portion of probasitarsus yellowish, in some cases profemur yellow. Hind leg dark brown, except posterior (and sometimes anterior) surface infused with yellow, sometimes entire metatrochanter and metafemur yellow. Tegula light brown, translucent. Pterostigma dark brown. Wings hyaline with dark setae. Dorsal surface of propodeum brown except basomedial triangle of green. **Metasoma:** Terga brown, except sometimes with basomedial patches of yellow. Metasomal sterna light brown to yellow.

***Pubescence.*Head:** Face with dull-white hairs (1–1.5 OD). Clypeus with long preapical fimbriae (2.5–3.5 OD). Gena with sparse, appressed hairs and long erect hairs (2.0–2.5 OD). **Mesosoma:** Pronotal lobe with tomentum on posterior margin. Mesoscutum and mesepisternum with sparse, off-white hairs (≤1.5 OD). Metanotum with long, erect hairs (2–3 OD). Mesofemur with sparse basal brush. Mesotibia with dense apical comb. Metafemoral scopa with long, plumose hairs. Metatibia with brown, plumose hairs (off white on posterior surface); basoventral hairs densely pectinate (almost palmate), directed apically. Metabasitarsus with brown hairs on anterior surface. Lateral surface of propodeum with long, sparsely-branched, plumose hairs (2.5–3.5 OD). Posterior surface of propodeum with short, appressed hairs. **Metasoma:** Terga sparsely pubescent; anterior surface of T1 with sparse, erect hairs (1–1.5 OD). Dorsal surface of T1 and T2 largely bare. Dorsal surface of T3–T5 with posteriorly directed hairs (1–2.5 OD), longer on T5 and laterally portions of (up to 3.5 OD). Ventrally reflexed portions of metasomal terga and metasomal sterna with long, sparsely-branched, plumose hairs (3.5–5.5 OD). Ventral hairs of T1–T2 directed medially.

***Surface sculpture.*Head:** Face granular. Clypeus with sparse, coarse punctures. Gena and postgena imbricate, shining. **Mesosoma:** Granular. Dorsal surface of propodeum (including metapostnotum) without raised sculpturing, posterior half smoother, imbricate. Lateral surface of propodeum imbricate, shining. Posterior surface of propodeum imbricate. **Metasoma:** Terga with punctation fine; dorsal surface of T1 and T2 impunctate, except along premarginal line. T1 and T2 dull, finely coriarious, apical impressed area of T2 and remaining tergal segments smooth, shiny.

**Figures 8–14.**
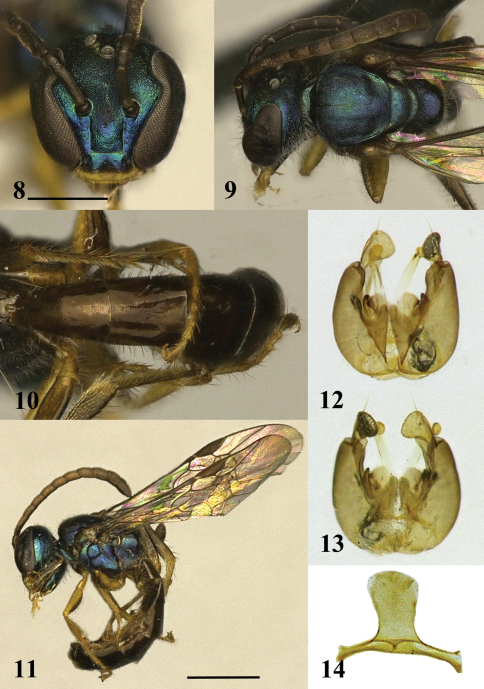
Allotype male of *Habralictus gonzalezi* sp. n. **8** Face, scale bar = 0.5 mm **9** Dorsal view of mesosoma **10** Dorsal view of metasoma **11** Lateral habitus, scale bar = 1 mm **12** Dorsal view of genital capsule **13** Ventral view of genital capsule **14** S7 and S8.

***Male.***(Figs 8–14)As in female except as follows. Body length: 4.3 mm. Head length: 1.00 mm. Head width: 0.96 mm. Forewing length: 3.2 mm.

***Structure.*Head:** Face longer than wide (length/width ratio = 1.04). Eyes strongly convergent below (UOD:LOD = 1.72). Eye emargination more acute. Clypeus weakly depressed medially. Mandible without preapical tooth. Labrum wider than long, without apical process. Supraclypeal area longer than clypeus (ratio = 1.18). Scape slender, extending to just below median ocellus. Pedicel subequal to F1. F2 and F3 subequal both about 2× length of F1. Flagellum clavate, extending beyond posterior edge of mesosoma. **Mesosoma:** Subequal in width to head (ratio = 1.02). Inner metatibial spur ciliate. Dorsal surface of propodeum (including metapostnotum) longer than mesoscutellum (ratio = 1.1). Lateral carina of propodeum fine, reaching half way to dorsal surface. **Metasoma:** Petiolate, T1 and T2 distinctly longer than wide. Metasomal terga with lateral portions weakly reflexed ventrally. Terminalia as illustrated (Figs 12–14).

***Colour.*Head:** Mostly blue-green. Labrum, mandible, and lower margin of clypeus yellow. Antenna dark brown, except ventral surface of flagellum orange-brown. Ocellar area pale green. **Mesosoma:** Blue-green, with pale green and purple reflections. Pronotal lobe brown. Fore leg yellow, except ventral surface of profemur brown with slight hint of metallic. Mid leg yellow, except anterior surface of mesofemur, mesotibia, and mesotarsus. Hind leg light brown, infused with yellow, except metatrochanter yellow. Dorsal surface of propodeum purplish. **Metasoma:** Brown, paler ventrally.

***Pubescence.*Head:** Clypeus with sparse, preapical fimbriae (2.5–3.5 OD). Gena with long erect hairs (2.5–3.5 OD). **Mesosoma:** Posterior surface of propodeum without short, appressed hairs. **Metasoma:** Terga sparsely pubescent; anterior surface of T1 with sparse, erect hairs (1–1.5 OD). Dorsal surface of T1 and T2 largely bare. Dorsal surface of T3–T5 with posteriorly directed hairs (1–2.5 OD), longer on T5 and laterally portions of (up to 3.5 OD). Ventrally reflexed portions of metasomal terga and metasomal sterna with long, sparsely-branched, plumose hairs (3.5–5.5 OD). Ventral hairs of T1–T2 directed medially.

***Surface sculpture.*Head:** Face granular. Clypeus with sparse, coarse punctures. Gena and postgena imbricate, shining. **Mesosoma:** Granular. Dorsal surface of propodeum (including metapostnotum) without raised sculpturing, posterior half smoother, imbricate. Lateral surface of propodeum imbricate, shining. Posterior surface of propodeum imbricate. **Metasoma:** Terga with punctation extremely fine, sparse; dorsal surface of T1 and T2 impunctate, except along premarginal line. T1 and T3 finely coriarious basally, apical impressed areas smooth, shiny.

##### Etymology.

The specific epithet is named for Victor H. González-Betancourt for his contributions to bee taxonomy and his encouragement and assistance with this manuscript.

### Key to species of Habralictus in the Lesser Antilles

**Table:** 

1	Antenna clavate, flagellomeres 11; metasoma petiolate, terga 7; (males)	2
–	Antenna not clavate, flagellomeres 10; metasoma ovoid, terga 6; (females)	4
2	Supraclypeal area and lower paraocular area dull due to imbricate microsculpture, punctures obscure to absent (Dominica)	*Habralictus gonzalezi* sp. n.
–	Supraclypeal area and lower paraocular area polished due to lack of microsculpture, punctures sparse but distinct	3
3	Profemur yellow ventrally; clypeal maculation nearly 1/2 clypeal length (Grenada)	*Habralictus insularis*
–	Profemur testaceous-brown ventrally with hint of metallic; clypeal maculation 1/3 clypeal length (St. Vincent and the Grenadines)	*Habralictus claviventris* (female unknown)
4	Clypeal punctures few, absent laterally; pronotal lobe brown; metatibia anterobasal hairs brown (Dominica)	*Habralictus gonzalezi* sp. n.
–	Clypeal punctures numerous, present laterally; pronotal lobe yellow; metatibia anterobasal hairs off-white (Grenada)	*Habralictus insularis*

## Discussion

The genus *Habralictus* is in need of taxonomic revision. *Habralictus gonzalezi* is only the 25^th^ described species in the genus (Table 1) but more undoubtedly remain to be described (Smith-Pardo 2009). The three species of *Habralictus* known from the Lesser Antilles are presumably derived from the South American species. Although, *Habralictus* is known to be distributed widely through the Neotropics no described species has been recorded from Venezuela, which is the closest area of the mainland (but see Ascher and Pickering 2011). The presence of *Habralictus* on Grenada, St. Vincent and the Grenadines, and Dominica makes it highly probable that the genus occurs on other islands in the Lesser Antilles, especially the islands St. Lucia and Martinique, which lie between Dominica and St. Vincent and the Grenadines.

**Table 1.** Checklist of world species of *Habralictus* Moure with known geographic distribution

**Name**	**Author**	**Distribution**
*Habralictus agraptes*	(Vachal 1904)	Bolivia, Peru
*Habralictus banghaasi*	(Schrottky 1910)	Bolivia
*Habralictus beatissimus*	(Cockerell 1901)	Brazil (Mato Grosso)
*Habralictus bimaculatus*^1^	Michener 1979	Colombia (Valle del Cauca)
*Habralictus callichroma*	(Cockerell 1901)	Brazil (Mato Grosso)
*Habralictus canaliculatus*^2^	Moure 1941	Brazil (Paraná, Rio de Janeiro)
*Habralictus chlorobaptus*	Moure 1941	Brazil (Goiás)
*Habralictus claviventris*	(Ashmead 1900)	St. Vincent and the Grenadines
*Habralictus crassiceps*^3^	(Moure 1941)	Brazil (Rio de Janeiro)
*Habralictus ephelix*	(Vachal 1904)	Bolivia, Peru
*Habralictus flavopictus*^4^	Moure 1941	Brazil (São Paulo)
*Habralictus grammodes*	(Vachal 1904)	Peru (Lima)
*Habralictus gonzalezi*	Gibbs sp. n.	Dominica
*Habralictus insularis*	Smith-Pardo 2009	Grenada
*Habralictus ligeus*	(Schrottky 1911)	Bolivia
*Habralictus macrospilophorus*	Moure 1941	Brazil (Rio de Janeiro)
*Habralictus manto*	(Schrottky 1911)	Bolivia (La Paz)
*Habralictus mapiriensis*	(Schrottky 1910)	Bolivia
*Habralictus metallicus*	(Friese 1916)	Costa Rica (San José)
*Habralictus orites*	Moure 1941	Brazil (Rio de Janeiro)
*Habralictus phacodes*	(Vachal 1904)	Bolivia, Peru
*Habralictus tradux*	(Vachal 1904)	Mexico (Chiapas, Jalisco)
*Habralictus trinax*^5^	(Vachal 1904)	Bolivia, Peru
*Habralictus xanthinus*	(Cockerell 1918)	Panama (Coclé, Panamá)
*Habralictus xanthogastris*	(Vachal 1911)	Colombia (Cundinamarca)

1. Nesting biology and sociality (Michener et al. 1979) 2. Nesting biology (Michener and Lange 1958) 3. Sole member of sometimes recognised subgenus *Zikaniella* 4. Type species of *Habralictus* 5. Senior synonym of *Augochlora maculiventris* Crawford, 1913

*Habralictus gonzalezi* is only the 18^th^ described species of bee recorded from the island of Dominica (Table 2). The bee fauna as currently known was almost entirely described by Crawford (1914; see also Moure et al. 2007; Table 2). Nearly twice as many species (31) are known from both St. Vincent and the Grenadines to the south and Puerto Rico to the North (Moure et al. 2007; Genaro and Franz 2008; Ascher and Pickering 2011). Other islands in the Lesser Antilles have even fewer recorded species than Dominica. This is includes Grenada which lies between St. Vincent and the Grenadines and mainland South America. It is likely that many more species of bees occur on these islands, particularly in the halictid fauna, for which several new Caribbean species have been described in recent years (Engel 2001, 2011a, 2011b; Genaro 2001). Two species of *Lasioglossum (Dialictus)* and one species of *Sphecodes* have also been examined from Dominica but description of these (if appropriate) will be done after a more thorough study of the West Indian species has been completed.

**Table 2.** Checklist of bee species recorded from the island of Dominica.

**Family**	**Species**	**Author**
APIDAE	*Anthophora footei*	Crawford 1914
	*Apis mellifera*^1^	Linnaeus 1758
	*Centris versicolor*	(Fabricius 1775)
	*Exomalopsis similis*	Cresson 1865
	*Melipona variegatipes*	Gribodo 1893
	*Melissodes rufodentata*	Smith 1854
	*Melissodes trifasciata*	Cresson 1878
	*Mesoplia azurea*^2^	(Lepeletier and Audinet-Serville 1825)
	*Xylocopa caribea*	Lepeletier 1841
	*Xylocopa transitoria*	Pérez 1901
HALICTIDAE	*Augochlora ignifera*	Crawford 1914
	*Habralictus gonzalezi*	Gibbs sp. n.
	*Lasioglossum punctifrons*	(Crawford 1914)
	*Lasioglossum* spp.	
	*Microsphecodes dominicanus*^2^	Stage 1972
	*Sphecodes* sp.^ 2^	
MEGACHILIDAE	*Coelioxys abdominalis*^2^	Guérin-Méneville 1844
	*Megachile concinna*^1^	Smith 1879
	*Megachile luctifera*	Spinola 1841
	*Megachile multidens*^1, 3^	Fox 1891

1. Exotic 2. Cleptoparasite 3. Possible junior synonym of *Megachile concinna* (see Genaro and Franz 2008).

## Supplementary Material

XML Treatment for Habralictus gonzalezi
